# Ultrasound Guidance for Botulinum Neurotoxin Chemodenervation Procedures

**DOI:** 10.3390/toxins10010018

**Published:** 2017-12-28

**Authors:** Katharine E. Alter, Barbara I. Karp

**Affiliations:** 1 Functional and Applied Biomechanics Section, Rehabilitation Medicine, Clinical Center, National Institutes of Health, Bethesda, MD 20892-1604, USA; 2 Combined Neurosciences IRB, National Institutes of Health, Bethesda, MD 20892-1604, USA; karpb@ninds.nih.gov

**Keywords:** botulinum neurotoxin, botulinum toxin, chemodenervation, guidance, ultrasound, electrical stimulation, electromyography, motor points, anatomic localization

## Abstract

Injections of botulinum neurotoxins (BoNTs) are prescribed by clinicians for a variety of disorders that cause over-activity of muscles; glands; pain and other structures. Accurately targeting the structure for injection is one of the principle goals when performing BoNTs procedures. Traditionally; injections have been guided by anatomic landmarks; palpation; range of motion; electromyography or electrical stimulation. Ultrasound (US) based imaging based guidance overcomes some of the limitations of traditional techniques. US and/or US combined with traditional guidance techniques is utilized and or recommended by many expert clinicians; authors and in practice guidelines by professional academies. This article reviews the advantages and disadvantages of available guidance techniques including US as well as technical aspects of US guidance and a focused literature review related to US guidance for chemodenervation procedures including BoNTs injection.

## 1. Introduction

The purpose of this invited article is to provide a review of ultrasound (US) guidance techniques for botulinum neurotoxin (BoNT) chemodenervation procedures including instrumentation, techniques and potential benefits and limitations of instrumented guidance with US.

BoNTs are approved by FDA and other regulatory agencies for the treatment of a diverse group of disorders including muscle over-activity (MoA) from various causes, neuro-secretory disorders and pain conditions [1,2,3,4,5,6]. The list of approved, proposed and investigational uses of BoNTs continues to expand at a rapid pace (Table 1).

The first step in evaluating a patient for BoNT therapy is obtaining a detailed history and physical examination followed by a functional assessment. This is to identify the severity, scope and impact of the problem or problems which are to be targeted by neurotoxin therapy as well as any co-morbidities, allergies, contraindications or other concerns that might influence treatment selection or dosing [7,8,22].

Patient assessment must include a detailed functional history as well as observing the patient performing a variety of tasks including activities of daily living and mobility (rolling, transfers, gait, stairs). This assessment will determine whether the potential target for BoNT therapy (muscle, gland, other) has a significant impact on the patient’s comfort, or passive or active function. Information provided by the patient, caregivers, therapists or other members of the health care team is critical for both identifying the problem as well as determining the goals of treatment. For example, when a patient is identified as having problematic spasticity associated with an upper motor neuron syndrome (UMNS), the clinician must evaluate whether MoA is problematic and thereby limiting comfort/care, passive or active function (reaching, grasping, transfers, walking etc.). Passive functional tasks are those performed for the patient by others (hygiene, dressing or other tasks). Active function includes tasks performed by a patient without assistance or with some assistance by others [7,8]. A comprehensive functional assessment also provides the clinician with useful information about which aspects of function are impacted by the various impairments and helps to identify which muscle or groups of muscles should be targeted for injection.

An important factor when considering BoNT therapy for a patient, particularly with UMNS related muscle over-activity (MoA) is the presence of other impairments. MoA is typically only one of several possible motor impairments and can be seen in patients with spasticity and/or dystonia. Other muscle impairments such as loss of selective motor control, weakness, and contractures can all contribute to the patient’s problem and/or a loss of function [9,10]. Prescribed in isolation, BoNT chemodenervation procedures may not fully address the patient’s impairments. For example, BoNT alone is unlikely to improve limited joint range of motion caused by MoA. To maximize the potential functional benefits of BoNT chemodenervation procedures clinicians typically recommend rehabilitation therapies including physical or occupational therapy, splinting, casting, orthotics and or adaptive equipment [7,8,22].

Once it has been determined that a patient may benefit from BoNT therapy, the next step is planning the treatment session. This includes selecting the muscles or other structures to be targeted. BoNT chemodenervation procedures require accurate localization of the injection target to enhance the efficacy and functional benefit from the procedure as well as to minimize the potential risks of these invasive procedures and possible side effects of the injected BoNT [22,23,24].

When choosing among the available targeting or localization techniques for BoNT injections, the following targeting goals should be considered;

- Accurately placing the BoNT within the target structure
- Avoiding structures in the path of the needle on its way to the target
- Avoiding inadvertent injection of structures adjacent to or behind the intended target

To achieve these targeting goals when performing BoNT injections, clinicians utilize one or more of the available guidance or localization techniques (Table 2). Traditional guidance techniques utilized by clinicians to guide BoNT procedures include;

- Manual needle placement using anatomic methods (surface/bony landmarks, range of motion (passive or active)) [22,24,25]
- Electromyography (EMG) [22,23,24,25]
- Electrical stimulation (E-Stim) [22,24,25]
- Image-based guidance (fluoroscopy, CT, MRI, ultrasound) [22,24,25]

Motor endplate targeting: Because BoNTs avidly bind to the presynaptic neuron at the neuromuscular junction, many clinicians also choose to use the published information about the location of motor points when choosing the injection site [11,12,13]. Motor endplate targeting is an add-on technique utilized in conjunction with manual, EMG, E-Stim or ultrasound localization techniques.

The primary focus of this review is the use of US guidance for chemodenervation procedures, with a brief mention of the importance of anatomy, the use of motor point localization techniques and the utility of combined use of US and electrophysiological techniques (EMG, E-Stim).

## 2. The Importance of Anatomy for Chemodenervation Procedures

When performing chemodenervation procedures including BoNT injections, no supplemental guidance technique can replace a clinician’s knowledge of anatomy [22,23,24,25]. This includes an in-depth familiarity with regional anatomy encompassing the location of muscles and adjacent structures (vessels, nerves, tendons, organs, and other structures). Also of critical importance is a detailed knowledge of cross-sectional anatomy, muscle origin/insertion/action and functional anatomy, i.e., the primary and/or secondary actions of muscles. This firm grasp of anatomy is the foundation upon which the procedure is planned, supplemental guidance techniques are added and the site for needle insertion is chosen.

## 3. Motor Point Localization

A number of anatomical and electrophysiological studies have mapped the location of motor endplates (MoEPs) in animal and human skeletal muscles [26,27,28,29,30,31]. Most MoEPs are reported to fall into one of several types of arrangements: a single innervation band, multiple innervation bands and, least commonly, a scattered or random distribution within the muscle [27]. BoNTs act by interfering with the function of soluble-N-ethylmaleimide-sensitive receptor (SNARE) proteins located in the cytosol of presynaptic cholinergic nerve terminals. By blocking the action of these polypeptides BoNTs prevent the exocytosis of synaptic vesicles and the release of acetylcholine. The resulting temporary chemodenervation reduces muscle contraction or activation in the injected muscle [1]. Based on the mechanism of action of BoNT a number of theoretical advantages and potential benefits of MoEP targeting have been suggested including reduced;

- Variability between treatment sessions
- Dose needed for efficacy
- Cost of the procedure (by reducing total dose)
- Potential reduction in spread to adjacent muscles or structures
- Risk of immune resistance

In a 2009 double blind RCT, Gracies et al. evaluated the impact of MoEP targeting and toxin dilution on the outcomes of BoNT injections for patients with spasticity in the biceps brachii muscle [32]. The authors concluded that either high-volume or endplate targeted BoNT (onabotulinumtoxinA) injections resulted in greater neuromuscular blockade, reduction in spasticity and co-contraction and improved active elbow extension range of motion than non-targeted or low volume injections [19]. Additional studies involving other muscle targets and in a larger number of patients who have muscle over-activity from various etiologies are needed to further assess the impact of MoEP targeting and or dilution on the efficacy and or potential adverse events following BoNT injections.

Van Campenhout et al. have published several studies on the location of MoEPs in lower limb muscles and on the effects of MoEP targeting on the efficacy of BoNT procedures in children with CP [31,33,34,35]. In a 2015 study, Van Campenhout et al. randomized children (4–18 years of age) with cerebral palsy receiving BoNT-A injections for treatment of spasticity in the gracilis muscle into 2 groups [34]. In both groups US was used to guide the injections and confirm needle location within the muscle. A fixed dose (in units/kg) and dilution of BoNT was used. The parents and children were unaware of which of the following injection techniques was utilized.

- In one group, BoNT-A was injected at a fixed proximal site 20–25% of the distance between the pubic symphysis and the medial epicondyle of the femur. Total doses 50 units or lower were injected at a single site. Doses over 50 units were equally divided into 2 injection sites placed 3 cm apart. The authors did not specify if the second injection was proximal or distal to the first site.
- In the second group, BoNT-A injection sites were determined by published information on the location of MoEPs in the gracilis muscle, based on either histological staining or anatomic dissections. The dose of BoNT-A was equally divided with ½ of the dose placed in the proximal 1/3 and the remaining units in the middle 1/3 of the muscle.

In this study, other muscles could also be injected with the non-randomized injections guided by US utilizing a MoEP targeting approach. 29 children (34 muscles) were randomized. Clinical spasticity outcome measures included the Modified Ashworth Scale (MAS) and Modified Tardieu Scale (MTS). A biomechanical/electrophysiological outcome measure, the Instrumented Spasticity Assessment (ISA) was also utilized. The ISA, which measures surface EMG and joint angular velocity, has been reported to be sensitive to change following BoNT injections.

The authors reported a greater reduction of spasticity on the ISA (surface EMG) in the gracilis muscles of children whose BoNT injections were based on MoEP localization compared to those whose received fixed-site injections. There was no difference between the 2 groups on clinical measures including the MTS or MAS. The authors concluded that injections of BoNT-A utilizing a MoEP injection technique are more effective in reducing pathological muscle activation or spasticity.

A 2013 study Van Campenhout et al. [35] compared MoEP guided injections into the iliopsoas muscle to more distal injections in the inguinal region). US was again utilized for muscle and needle localization. Outcome measures included pre- and post-injection atrophy, assessed by volume calculated from 3T MRI, in the psoas muscle as a measure of BoNT effect. The authors reported iliopsoas atrophy in the children injected utilizing a MoEP targeting technique (muscle volume decreased to 79% of pre-injection volume) compared to the distal site injections (post injection muscle volume 107% of pre-injection volume). Thus, injections utilizing MoEP targeting of the psoas muscle appeared to be more effective than injections placed distally.

These reports highlight the need for additional studies to evaluate the impact of MoEP targeting for spasticity as well as for other indications, and especially to assess effects of different targeting approaches on functional outcome measures such as gait.

## 4. Selection of Guidance Techniques

A number of factors influence a clinician’s choice of guidance for BoNT chemodenervation as with other procedures including: the clinician’s training and expertise, availability of equipment, advantages/limitations of a given technique, the target for injection and patient-related factors (condition/diagnosis, size, cooperation, presence of contractures/deformity, and others) [23,24,25,36]. Recognizing the potential advantages and limitations of each of possible techniques is key when selecting a targeting method (Table 2).

## 5. Ultrasound Guidance for BoNT Injections

While high frequency US guidance is a relatively recent addition to the clinicians’ tool box for BoNT injections, it has been used for decades to guide a wide variety of invasive procedures including biopsies, regional anesthesia and diagnostic and neurolytic nerve blocks [36]. US combined with E-Stim is widely used by anesthesiologists who perform regional anesthesia and has been found to decrease the procedure time, volume of injectate required to produce a block, time for block onset and the quality of the resulting block [37]. Since the first reported use of ultrasound to guide BoNT injection in 1996 for treatment of achalasia, the number of studies and case reports of US to guide BoNT when treating patients with spasticity, dystonia and pain conditions continues to grow [38,39,40,41,42,43,44,45].

A potential barrier or limitation to the use of US is that although recent physician graduates of medical school or post-graduate training programs may have received training in the use of US, many, if not most clinicians and trainees, have not received this training. Access to the necessary training and gaining expertise in the application of US for chemodenervation procedures requires a significant commitment of time to acquire the skills necessary to incorporate US into clinical practice. The training required to become proficient in US guidance for BoNT procedures includes didactic and hands-on courses, review of published materials and techniques followed by months of self-study and scanning practice [46,47]. Hands-on practice is critical to acquire the necessary skills including equipment use, US pattern recognition and the motor skills required to coordinate simultaneous scanning and injections. While this process may at first seem daunting, the necessary skills are attainable with a commitment to training and practice.

## 6. US Imaging, Physics and Technical Review

Of the available modes of ultrasound imaging, Brightness or B-mode US is the mode typically used for procedural guidance. The B-mode provides direct visualization of the depth/position of the target structure, the safest path to the target, continuous visualization of the needle on its path to the target and of the toxin/injectate spread within the target [36,47,48]. Direct/continuous US visualization, enables the clinician to redirect the needle so that its trajectory can be adjusted to avoid nerves, vessels, tendons or organs. Color and power Doppler imaging are also useful when performing BoNT injections. These techniques discriminate flow in vessels and therefore are especially helpful to identify vessels in the path to the target as well as to discriminate vessels from adjacent nerves when performing neurolytic procedures [24,25,47,48,49].

The basis of US imaging and guidance is the production and transmission of sound waves of various frequencies, transmitting these soundwaves to the patient’s tissues and processing the returning waveforms to create grey scale images and cine-loops (videos). Soundwaves are produced by piezoelectric crystals which are embedded into a US transducers and connected to the US machine via a wire/electrical cable [24,47,48].

Piezoelectric crystals convert the electrical pulses generated by the CPU unit of the US machine into mechanical vibrations in the head of the transducer. When the transducer is placed in contact with the patient, the soundwaves are transmitted to the patient. A coupling agent (gel, water) is typically used to decrease skin impedance and thereby enhance sound transmission [24]. Transmitted soundwaves are either absorbed, transmitted, reflected or refracted off of various tissue interfaces in the body. The soundwaves reflected back to the transducer are then converted back into electric pulses by the piezoelectric crystals, are returned to the CPU unit of the US machine, and are processed into grey-scale images (using a time distance coefficient and other processing techniques). A full discussion of US physics and equipment is beyond the scope of this chapter and the reader is referred to several reviews on this topic [24,47,48].

The variation in the grey-scale image obtained with B-mode US is determined by the number of echoes returning to the transducer, which in turn is determined by the sonoacoustic properties of a tissue. When imaging with B-Mode US, tissues are described by their echo-texture or sonoacoustic appearance. Structures which are highly reflective of sound waves will appear bright or hyperechoic. This is because this tissue or structure is highly reflective of sound waves reflecting many or most of the sound waves back to the transducer. Conversely, structures will appear dark or hypoechoic when few (or relatively few) US waves are reflected back to the transducer. Fibro-fatty connective tissue fascia and bone appear hyperechoic. Tissues with a higher water content will appear hypoechoic or relatively hypoechoic. Mirror-like tissues or structures (bone, metal implants) reflect all soundwaves back to the transducer; no soundwaves penetrate the structure. This results in an anechoic shadow which is completely black or devoid of echoes [24,47,48,50].

**Figure 1:**
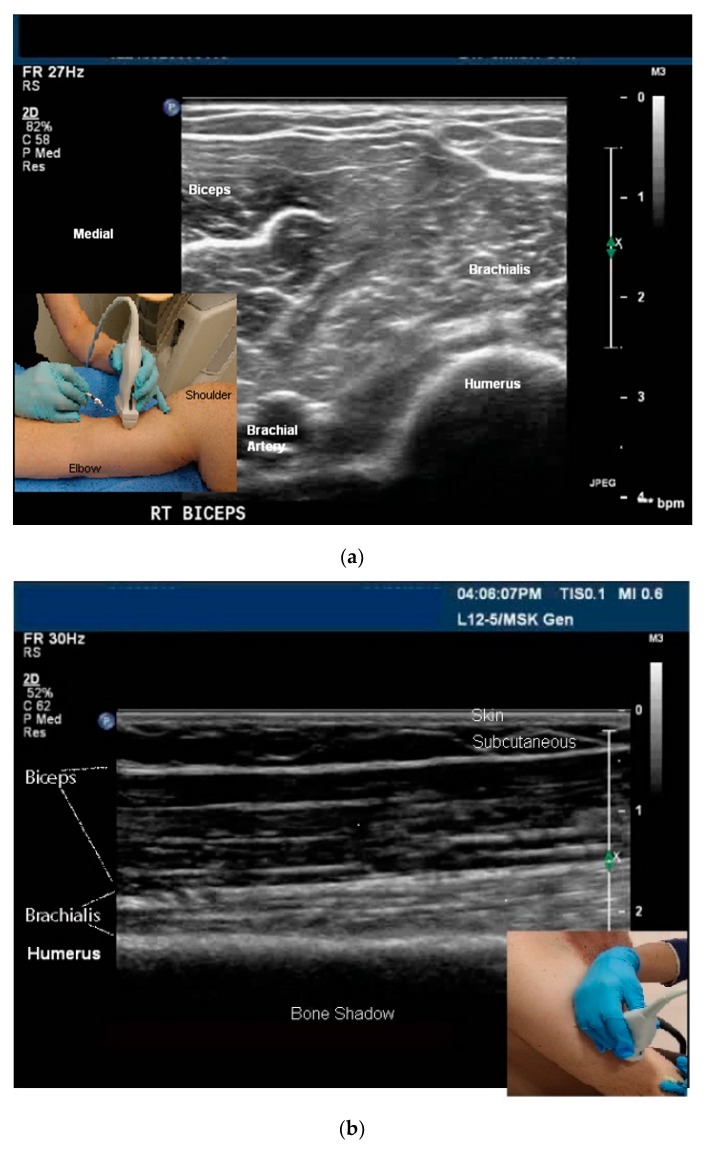
(**a**) Transverse B-mode image, dDistal 1/3 Arm, anterior/flexor muscles; (**b**) longiudinal B-mode image, distal 1/3 Arm, anterior/flexor muscles.

**Figure 2:**
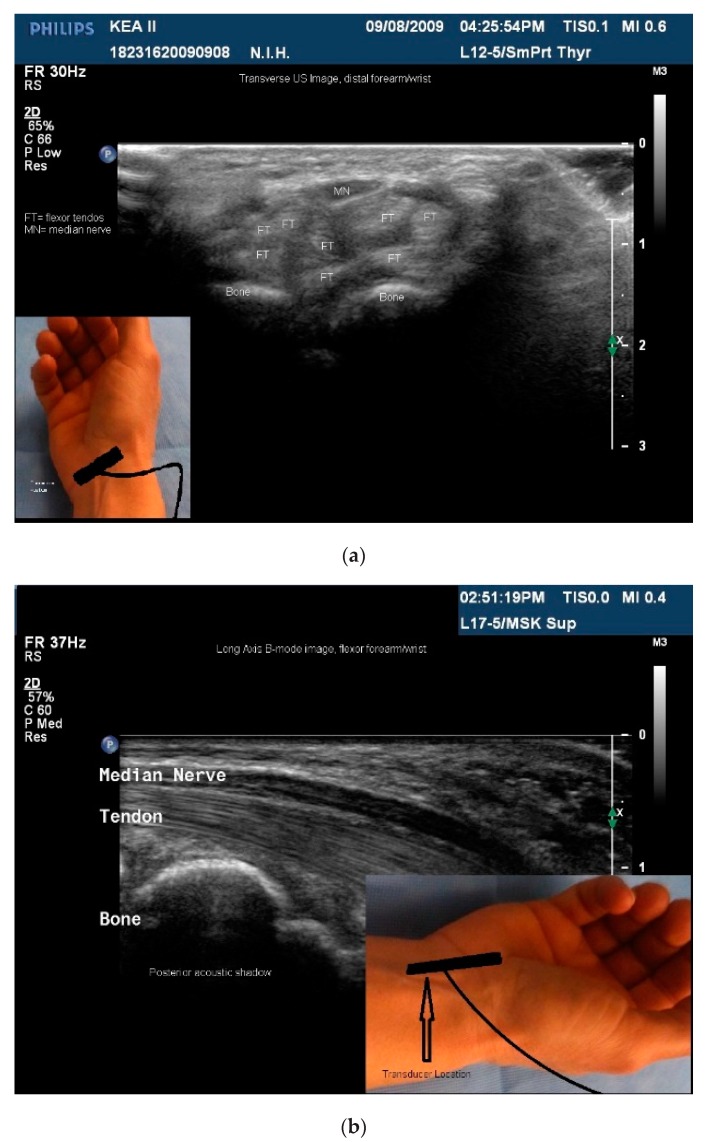
(**a**) Transverse B-mode ultrasound (US) image, distal flexor foream/wrist; (**b**) longaxis B-mode US image, distal flexor foream/wrist.

**Figure 3:**
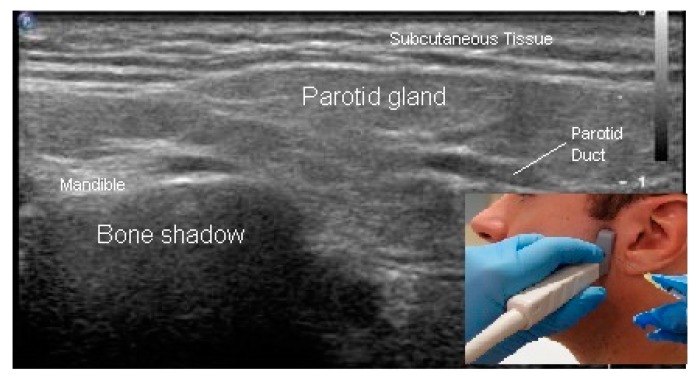
B-mode ultrasound image, parotid salivary gland.

Sonoacoustic Characteristics of Relevant Tissues:

- Skeletal muscle: The sonoacoustic appearance of muscle is a mix of hyperechoic intramuscular connective tissue and hypoechoic contractile fascicles. In long axis or longitudinal view, the contractile elements and connective tissue of skeletal muscles appear as hypoechoic or hyperechoic linear bands or streaks. In transverse view, the muscle has a speckled hypo/hyperechoic appearance from the mix of contractile fascicles and connective tissue viewed in short axis [47,48,49,50]. Figure 1a,b.
  ⚬ Caveat: The sonoacoustic property of muscle may be altered by longstanding spasticity. Due to atrophy of and/or fibro-fatty replacement of the normally hypoechoic contractile elements, muscles may appear hyperechoic. This is most notable in patients with more severe impairments and functional limitations [24]
- Tendons: are hyperechoic, highly anisotropic and fibrillar in appearance [47,48,49] (Figure 2a,b).
- Nerves: are less hyperechoic or fibrillar than tendon. With a high frequency transducer (≥12 mHz), nerves fascicles can be visualized. In longitudinal view, the epineurium will appear hyperechoic compared to the relatively hypoechoic nerve fascicles giving the nerve the appearance of a “railroad track”. In transverse view the relatively hyperechoic epineurium surrounds the hypoechoic fascicles giving the nerve a speckled appearance [49,51] (Figure 2a,b).
- Blood vessels are anechoic (black) [48,49] (Figure 1a).
- Bone is mirror-like and highly reflective of US. Therefore, the cortex of bone will appear hyperechoic and, since no US penetrates the cortex, posterior acoustic shadowing will be observed [47,48,49] (Figure 1a,b and Figure 2a,b).
- Glands, including salivary glands, have a uniform grey-scale appearance [49] Figure 3.

## 7. Equipment

The equipment required to perform US-guided chemodenervation procedures includes [24,48,52].

**Figure 4:**
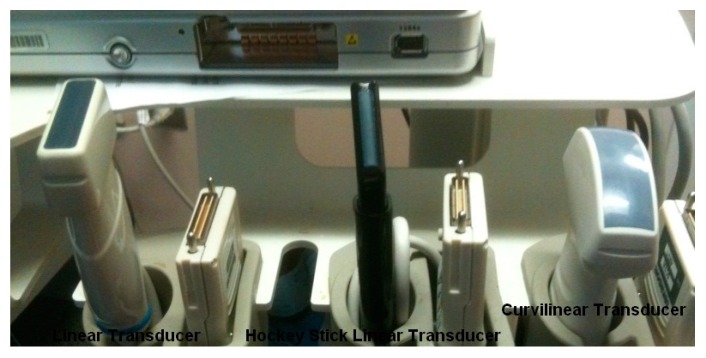
Ultrasound transducers.

- A US machine
  ⚬ The majority of machines come with factory-installed musculoskeletal, nerve and/or glandular imaging presets and needle guidance software all of which speed system navigation, optimize imaging of the structures of interest, thereby enhancing efficiency of the procedure.
- Transducers of various frequencies, sizes and shapes, see below (Figure 4).
- Manufacturer’s approved transducer cleaners/disinfectants.
- A coupling agent such as ultrasound gel to reduce skin impedance.
- Procedural supplies: gloves, skin disinfectant, needles of various lengths and transducer covers if desired/required.

Selection of the correct transducer is key to the success of US imaging and its use for procedural guidance including chemodenervation procedures [24,48,49,53]. Transducer characteristics need to be matched to the territory being scanned, including:

- Frequency: The frequency of the soundwaves produced by a transducer determines both the depth of penetration as well as image resolution. High frequency sound waves lead to images of greater resolution. However resolution is at the expense of depth of penetration because soft tissues absorb high frequency soundwaves leaving few or no waveforms to travel on to image the deeper tissues. Adequate imaging of deeper tissues (including muscles) requires a transducer which emits lower frequency waveforms. Most commercially available transducers emit a range of frequencies, for example 17–5 MHz, 12–5 MHz, 5–2 MHz, etc. [52] (Table 3).
  ⚬ Transducers which emit sound waves with a frequency ≥12 MHz are considered high frequency transducers and are typically used for cervical, upper limb, and some lower limb muscles and for pediatric patients [52].
  ⚬ Ultra-high frequency transducers (≥22 MHz) are now available; one manufacturer has transducers that produce 48–70 MHz soundwaves. These instruments provide exquisite imaging of superficial structures including nerves, vessels and superficial vessels. This equipment is currently being used by some clinicians for diagnostic nerve imaging, vascular access and chemodenervation injections of superficial muscles in the face and hand [53].
  ⚬ Lower frequency transducers used for musculoskeletal imaging range from 3–5 MHz; mid-range frequency transducers are from 7–10 MHz [52].
- Size/Shape: Small footprint linear transducers including hockey stick transducers are useful for irregular surfaces such as the hand or face or for small pediatric patients. Curvilinear transducers (which are typically lower in frequency) are used when imaging deeply seated muscles or structures (Figure 4). Specialty transducers, such as endocavitary transducers, may be used for some BoNT injections, for example into prostate, vagina, or bladder [24,52].
- US Beam Width: The width of the US beam created by the piezoelectric crystals/transducer is less than the width of a credit card. The width of the US beam should not be confused with the width/footprint of the transducer itself [24,52,53].

## 8. US Scanning Techniques

The first step in identifying structures for procedural guidance is to perform a preliminary or “scout” scan of the region of interest. This scan provides clinicians with detailed information about the entire region of interest including the target depth and location, adjacent structures, optimal path to the target and structures to be avoided (such as nerves, vessels and organs). Because the US beam produced by a transducer is narrow, only a thin slice of tissue is visualized when the transducer is placed on the patient. To completely image both the region of interest and the target requires the transducer be systematically moved proximally and distally and side-to-side [24,45,49,50,51,52,53]. The transducer is typically rotated to image muscles in both a true transverse and a longitudinal view. This may require an oblique orientation of the transducer on the body due to the orientation of the muscle of interest.

**Figure 5:**
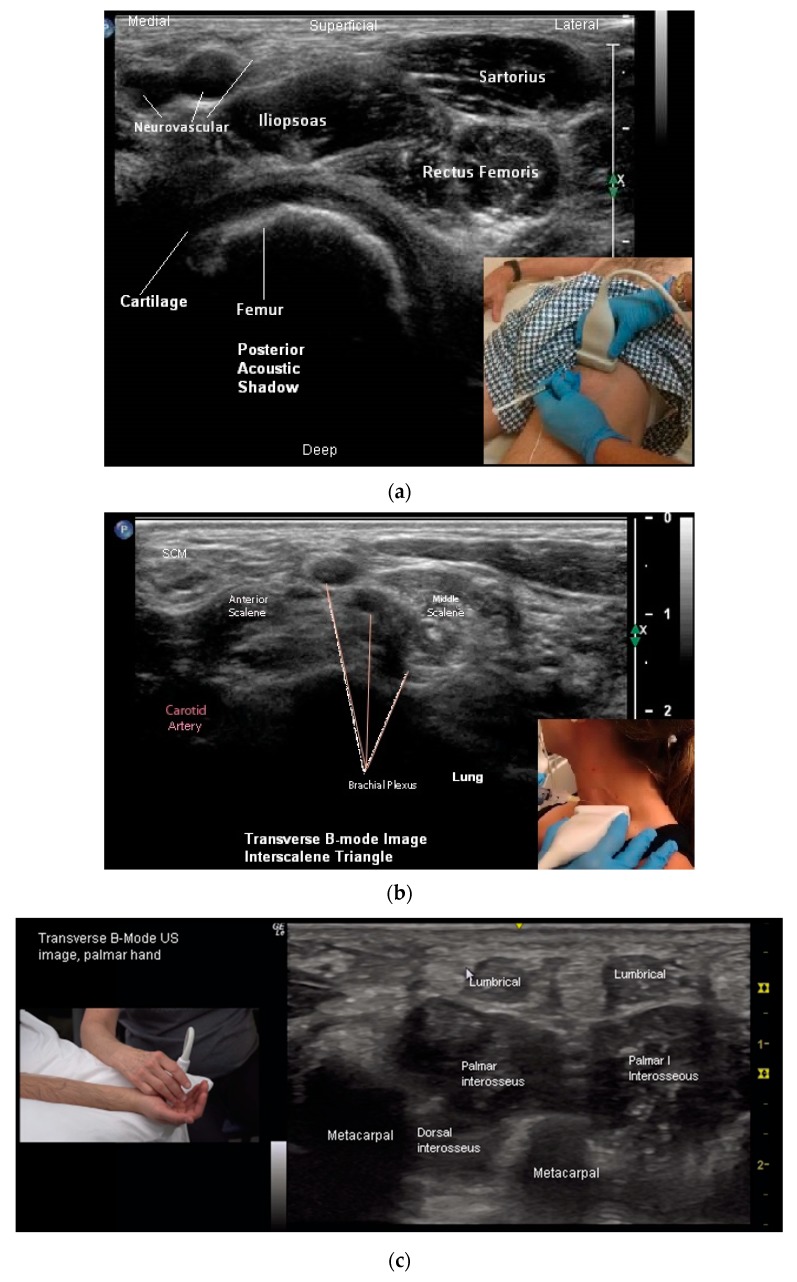

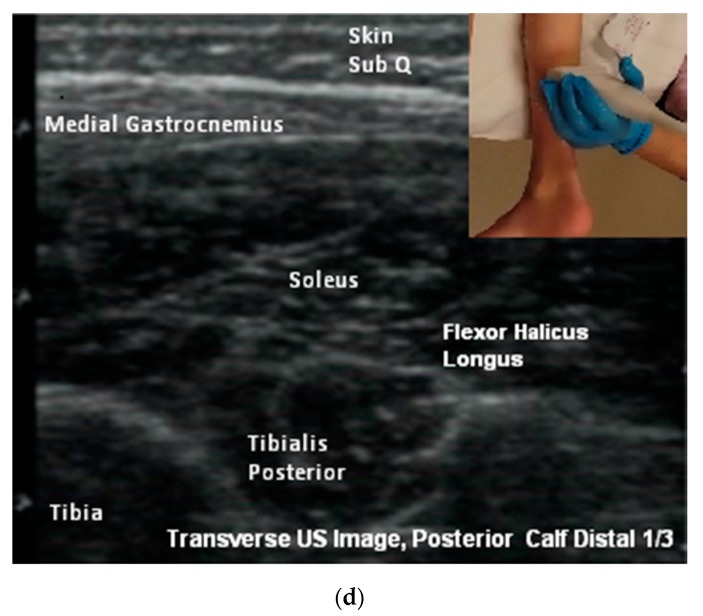
(**a**) Transverse B-mode US image, Proximal Thigh; (**b**) transverse B-mode US image, Interscalene Triangle; (**c**) transverse B-mode US Image, hand (palmar view); (**d**) transverse B-mode US image, posterior calf (Distal 1/3).

**Figure 6:**
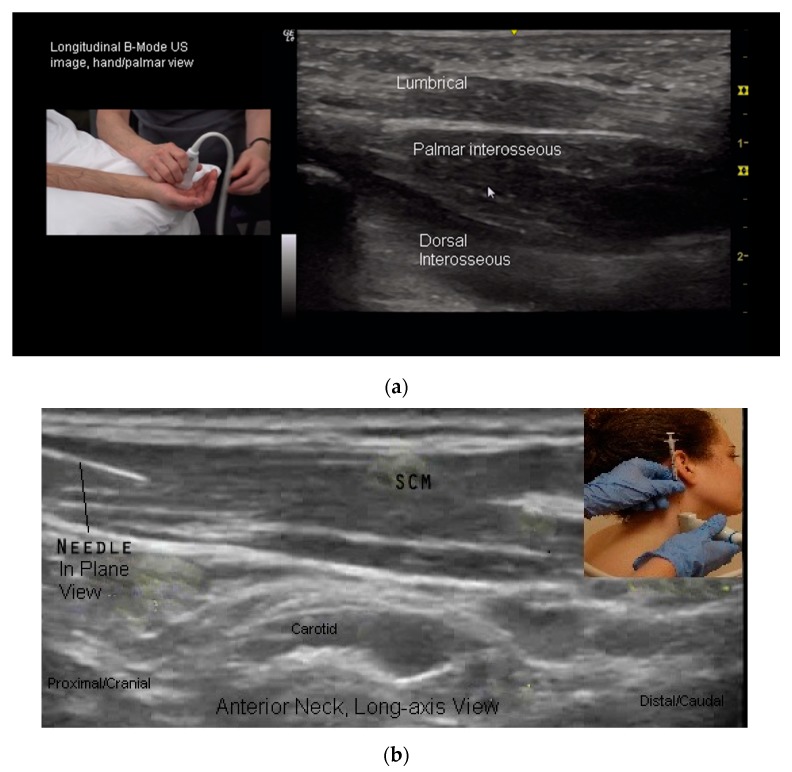

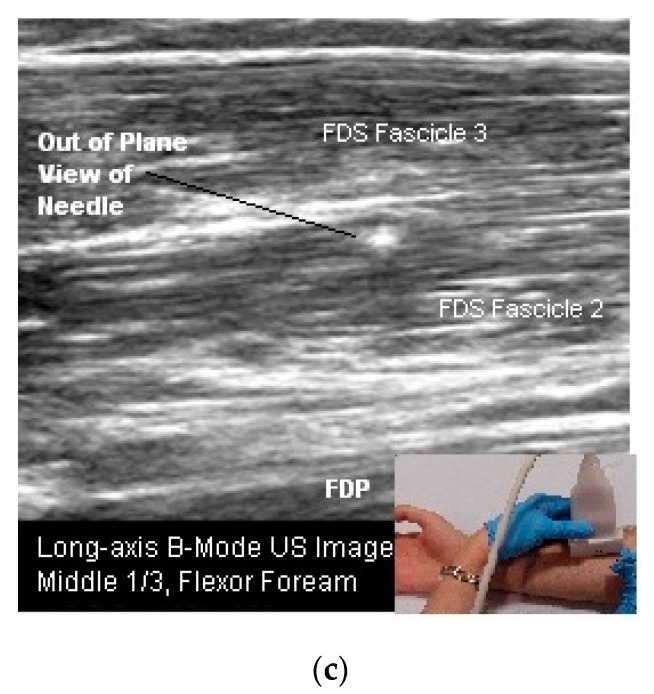
(**a**) Long-axis B-mode US image, hand (palmar surface); (**b**) long-axis B-mode US image, anterior neck; (**c**) long-axis B-mode US image, flexor forearm.

**Figure 7:**
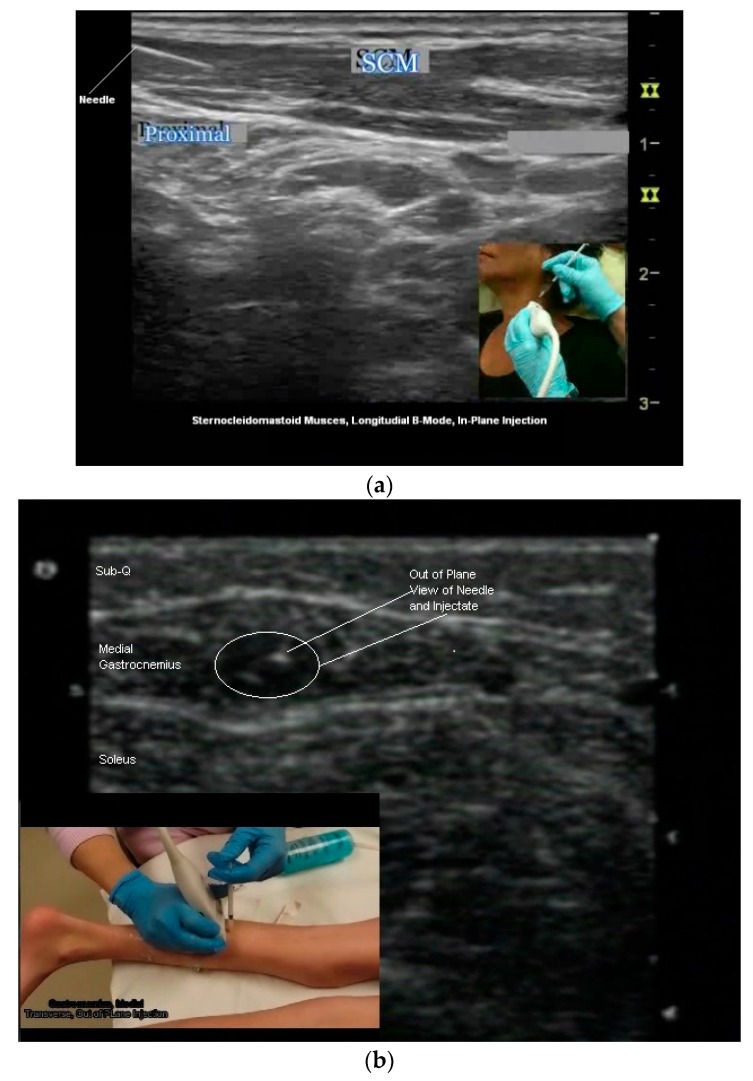

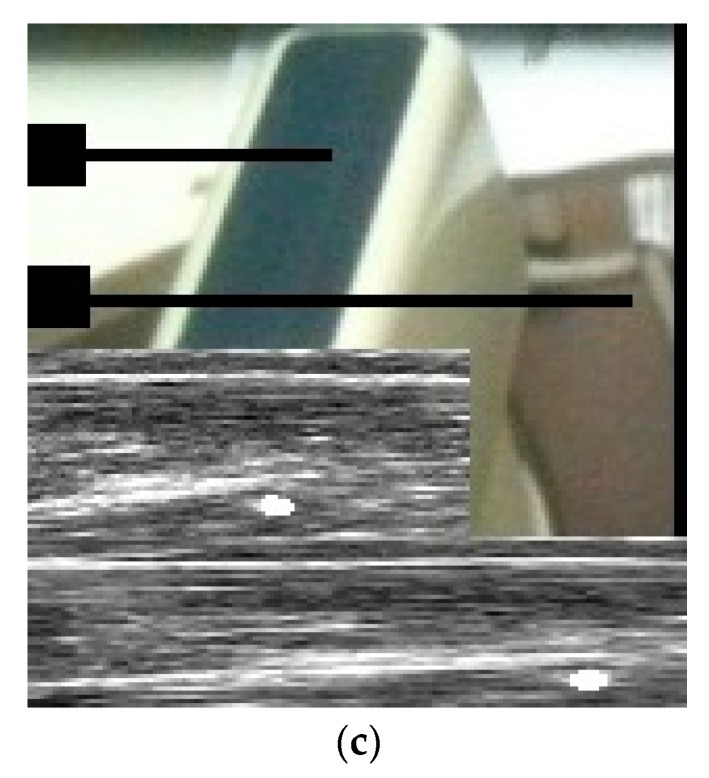
(**a**) Longitudinal B-mode US image, inplane injection; (**b**) transverse B-mode US image, out of plane injection medial gastrocnemius; (**c**) illustration, out of plane view of needle tip and shaft.

While muscles can be imaged in either transvers or longitudinal view, muscle pattern recognition is best obtained in the transverse view [24] (Figure 1a,b, Figure 5a–d, Figure 6a–c and Figure 7a,b). Features that are better appreciated in the transverse view muscles include muscle contour lines and adjacent muscles, bones, vessels, nerves and other structures [24,45,49,53] (Figure 1a and Figure 5a–d).

## 9. US Guided Procedures Techniques

The muscles to be targeted and guidance technique(s) for a chemodenervation are determined by the treatment plan, which is in turn determined by the patient assessment [7,8,22]. The targets for injection determine transducer selection, patient positioning and the procedure setup. For head and neck injections, most patients are positioned in a sitting position, partially reclined. For upper limb injections, either a sitting, supine and or side-lying position will provide access to most muscles. For lower limb muscles the patient should be positioned prone for lumbar paraspinal, gluteal, posterior thigh and calf muscles or supine for anterior thigh/hip and leg muscles.

When using US guidance, muscles, glands, nerves and tendons are identified and distinguished from one another based on their sonoacoustic properties. Individual muscles are further identified by using pattern recognition including contour lines and adjacent structures, typically in transverse scanning mode. Passive or active range of motion (either in transverse or longitudinal view) may also be used to help identify muscles and individual muscle fascicles [24,49,50,53] (Video S1).

Once the injection target, site for needle insertion and the safest path of the needle to the target are identified, the needle is inserted through the skin and advanced to the target under continuous visualization [24,45,49,50,54]. When the needle is visualized in the target, the clinician then injects the toxin (following aspiration of the syringe). During injection, the volume of injectate within the target and volume distension can be assessed and the needle can be repositioned within the target if needed. The needle is withdrawn and, if multiple injection sites, are planned then reinserted a new site repeating the process described above.

Two needle insertion techniques are utilized when performing US guided BoNT injections: Out of plane and in plane (Figure 6b,c and Figure 7a–c). There are advantages and limitations to each insertion technique. One technique may be superior to the other for a given patient or particular muscle/structure. Therefore clinicians should be familiar with both approaches [24,50,54].

**Figure 8:**
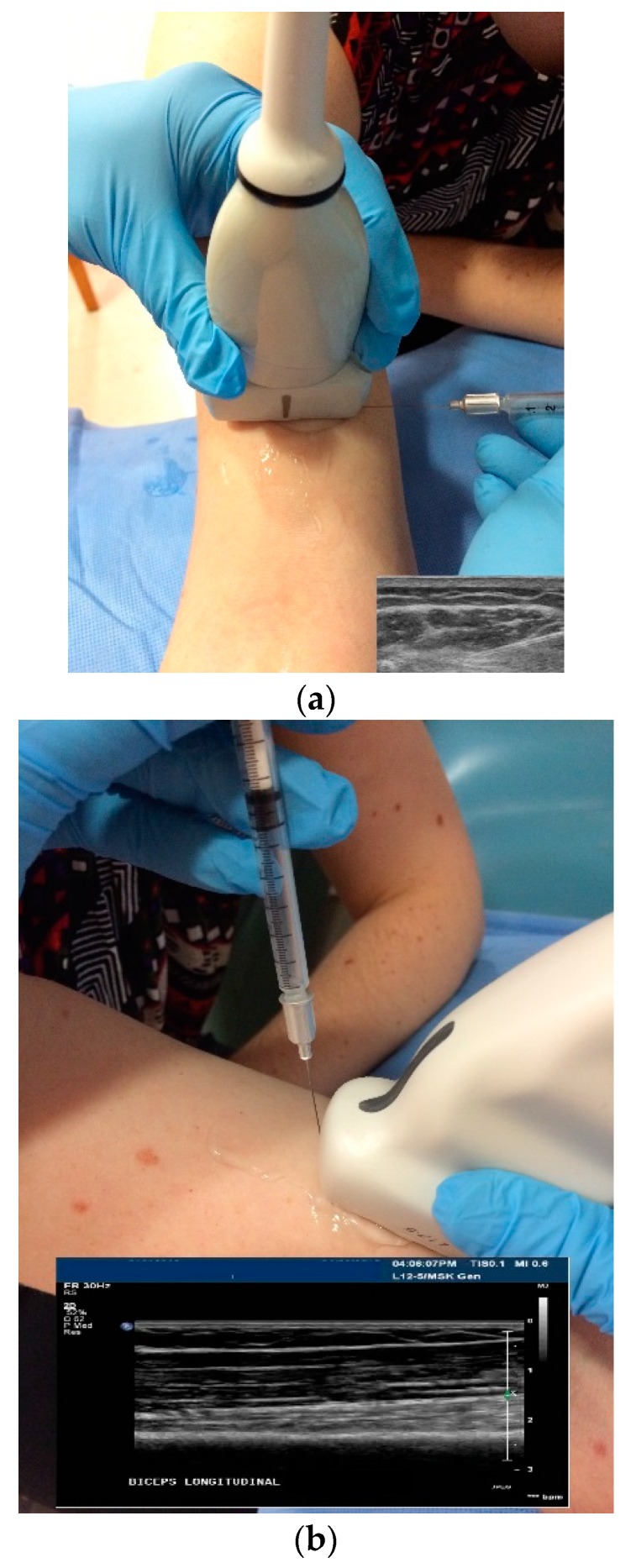
(**a**) Flat angle of needle insertion using an in plane technique, effect on needle visualization. Needle is visualized; (**b**) steep angle of needle insertion using an in plane technique, effect on needle visualization. Visualization is lost due to anisotropy.

- Out of Plane Technique (OPT): When using an OPT, the needle is inserted across the short axis of the transducer. Using this technique, a cross sectional view of the needle is obtained and the needle is visualized as a hyperechoic dot [34,50,54] (Figure 6c and Figure 7b,c).
- Potential advantages of the OPT: This technique often provides the most direct path to the target.
- Potential limitations of OPT: The primary limitation of the OPT is that only a cross-sectional view of the needle is seen. Therefore the entire length of the needle is not visualized.
- When using an OPT it is critical to keep the tip of the needle within the US beam. If the needle tip is inserted beyond the US beam, it cannot be visualized and may be in an untargeted structure. To track the path of the needle to the target, a “walk-down” technique is utilized. With this technique, the needle is jiggled up and down in very small movements and advanced slowly to the target as the clinician observes the movement in the pertubated tissues as the needle is inserted from superficial to deep. When the needle tip reaches the target, a small quantity of the injectate is injected into the target and can be seen on US, confirming the correct location [24,50,54]. The remainder of the injectate is then injected.
- In Plane Technique (IPT). When using an IPT the needle is inserted along the length of the transducer (Figure 6b and Figure 7a). With the IPT, the entire needle and its tip is visualized, an advantage over the OPT. However, this technique can be challenging to perform because the sonographer must keep the needle within the narrow US beam. Another challenge is that optimal needle visualization requires that the needle be inserted and maintained in an orientation perpendicular to the US beam Figure 8a. When inserted at a steep or oblique angle, visualization of the needle may be lost due to needle anisotropy [24,50,54] (Figure 8b).

## 10. Ultrasound Artifacts

- Anisotropy is one of several important artifacts encountered during US imaging, others being posterior acoustic shadowing, posterior acoustic enhancement, and reverberation. Anisotropy is a characteristic of some tissues/structures (like tendons) and of needles, whereby the reflection of soundwaves from the tissue or structure is affected by the incidence angle of the soundwaves. When soundwaves are perpendicular to the structure, the structure can be visualized in its entirety but visualization of the structure is compromised or lost at other angles [50,54,55,56,57].
  ▪ To keep the needle perpendicular to the US beam requires needle insertion to be at a flat angle relative to the transducer (Figure 8a). For superficial structures, such as the sternocleidomastoid muscle, this is easily accomplished. When targeting deeper muscles/structures, the needle must be inserted at some distance from the transducer to maintain an angle that minimizes anisotropy. This often requires a larger gauge, longer needle.
  ▪ The oblique approach may also be problematic when targeting some structures (such as anterior or middle scalene muscles) where an indirect path to the target may traverse regions with large vessels and nerves which must be avoided Figure 5a,b.

## 11. Combined Guidance Techniques

Ultrasound may be used in conjunction with the electrophysiological guidance techniques of EMG or E-Stim.

⚬ Combined EMG + US guidance is frequently helpful when treating patients with complex postures associated with cervical dystonia, limb dystonia or other conditions where multiple muscles may contribute to the same posture or problem. In this case, an EMG amplifier or instrument and an EMG injection needle are used during US guidance. US provides accurate anatomical localization of the needle electrode while EMG provides information about the activity of the muscle and whether there is tonic activity suggesting its contribution to the observed abnormal posture. EMG may also be used to for localization of motor points which performing neurolytic chemodenervation procedures [24,57]. (Video S1).
⚬ E-Stim + US is typically used for diagnostic, neurolytic or anesthetic nerve blocks and/or motor point blocks. The injection needle electrode, which is used the stimulator setting on an EMG machine or hand-held stimulator, is inserted under US guidance and advanced to a near-nerve location [24,37,55,56]. The stimulator is turned on and muscle twitch is observed. The needle position is adjusted or advanced while reducing the intensity of the stimulation until an optimal sited is located; the agent is then injected through the same needle. Current US technology cannot visualize motor points for motor point blocks but US can be used to accurately insert the needle into the selected muscle and the needle position adjusted using the E-Stim or EMG to identify the MoEPs. US guidance alone is not used in isolation for nerve or motor point blocks [24,55] (Video S2).

## 12. Guidance Methods for BoNT Injections: Levels of Evidence

### 12.1. Systematic Reviews

Chan et al. in 2017 published the results of a systematic review of the existing literature evaluating the impact of injection technique on outcomes of BoNT procedures for limb spasticity. Their search revealed 597 studies 9 of which met the inclusion criteria (English language, randomly controlled trials (RCT) from 1990–2016, studies with unrestricted injection methods, comparison of at least two injection techniques, similar BoNT dose, patients ≥16 years) [58]. The injection techniques were categorized into four types: (1) Localization technique; (2) site selection; (3) injectate volume; (4) injectate volume plus site selection.

Based on their review the authors concluded that there is Level 1 evidence that the following techniques are associated with improved outcomes when performing BoNT injections for limb spasticity;

⚬ Instrumented guidance (US, EMG, ESTIM) were found to be superior to manual needle placement
⚬ Injections targeting MoEPs were superior to multisite quadrant injections
⚬ Injections targeting MoEP injections is equivalent to multisite injections
⚬ High volume injections are similar to low volume injections
⚬ High volume injections distant to the MoEP are more effective than low volumes near the MOEP

Additional conclusions:

⚬ While this systemic review did not specifically compare the various instrumented techniques to one-another one reviewed study reported that US and E-Stim were equivalent and both were superior to manual guidance for localization of the wrist and finger flexors [59].
⚬ The author’s reported that only five of the nine the studies were powered for statistical significance limited conclusions when the study’s comparison of techniques was negative.
⚬ Based on their review the authors concluded that US and E-Stim are superior to manual needle placement but that additional studies, involving a larger number of subjects are needed to evaluate functional outcome with the various guidance techniques.

Grigaui et al. reported the results of a systematic review of the impact of guidance technique on the outcomes of BoNT injections for spasmodic torticollis and spasticity in adults and children [60]. The authors concluded that there is strong, Level 1 evidence to support the superiority of instrumented guidance for BoNT injections (EMG, E-Stim or US) compared to manual needle placement for patients withspasmodic torticollis, upper limb spasticity, post stroke spastic equinus and spastic equinus in children with cerebral palsy. No specific recommendations were made regarding the choice of instrumented guidance other than US was reported to be more effective than ES for spastic equinus in adults with post stroke spasticity.

BoNTs, when injected for muscle over-activity, are reported to reduce pain in a variety of conditions including cervical dystonia [10]. BoNTs are also are also utilized by clinicians, for the treatment of a variety of pain conditions, see Table 1. A review of the literature identified no systematic reviews of studies which compared the accuracy or efficacy of two or more guidance methods for BoNTs specifically for pain indications/conditions. There are a number of systematic reviews of US guidance for a variety of musculoskeletal (MSK) injections or procedures for pain [36,61,62]. These reviews compared the accuracy and or efficacy of blind or landmark guided injections to those guided by US for variety of musculoskeletal conditions including knee arthrocentesis, shoulder or knee joint injections for pain and pain associated with subacromial bursitis. In all of these reviews US guidance was reported to be associated with greater reduction in pain and improved functional outcome when compared to relying on landmark based or blind guidance.

### 12.2. Other Articles

Injection Pain: Reducing pain associated with BoNT injections is important as many patients, particularly children, report significant discomfort with injections. In addition, the pain associated with EMG or E-Stim guidance in children may require general sedation which increases the risks and costs of chemodenervation [63,64,65]. There are anecdotal reports that US guidance for BoNT injections is associated with less pain when compared to injections guided by EMG or E-Stim [65]. This finding was confirmed by a 2014 prospective study which reported the impact of guidance technique (E-Stim or US) on pain for lower limb muscle injections in children with spasticity (Bayon-Mottu M). Pain was evaluated immediately post-procedure using a visual analog scale and a behavior based scale (FLACC (Face, Leg, Activity, Cry, Consolability). Children and or their caregivers reported a significantly lower level of pain with procedures guided with US compared to those with E-Stim guidance [66].

Accuracy of Needle Placement and Procedure Time: US has also been reported to increase the accuracy and the speed of BoNT procedures in children.

⚬ Berweck and colleagues reported over 6000 US guided injections in 70 muscles in 350 children from 2000 to 2003. The authors reported that the time to identify and perform injections under US guidance in the target muscles varied from five s in superficial muscles to 30 s in deep muscles. They also reported that US permitted nearly all muscles to be accurately and quickly targeted, even when anatomic muscle variations associated with patient’s age or size, or level of disability were encountered. The authors also noted that patient cooperation for muscle recruitment was not required for US guided injections [44].
⚬ Chin and colleagues [42] evaluated the accuracy of manual needle insertion (subsequently checked by E-Stim or US) for upper and lower limb muscle injections in children with cerebral palsy. They reported that the accuracy of manual placement was acceptable (>75% accuracy) only in the gastroc-soleus. Accuracy of needle placement was reported to be unacceptable for a range of other lower and upper limb muscles, including hip adductors (67%), medial hamstrings (46%), tibialis posterior (11%), biceps brachii (62%), forearm and hand muscles (13% to 35%). Based on the results of this study, the authors recommended the use of electrical stimulation or other guidance techniques for needle placement in all muscles excepting gastroc-soleus [43].
⚬ Yang and colleagues reported that accuracy using manual needle placement (checked by US) for BoNT procedures in the medial gastrocnemius was relatively high (92.6%). However, they reported that the accuracy of needle was significantly lower in the lateral gastrocnemius, ranging from 57% in younger children to 64.7% in the group as a whole. The authors concluded that this limited accuracy was likely due to smaller size or thickness of the lateral head of the gastrocnemius when compared to the medial head [41].

Comparing Adverse Events with Various Guidance Methods: Reducing adverse events or side effects associated with BoNT is important for all conditions and all guidance methods.

⚬ Hong et al. compared the incidence of dysphagia in patients treated for cervical dystonia using EMG versus US. The incidence of dysphagia was 34.7% in patients where injections were guided by EMG alone. When the patients with dysphagia were converted to receiving BoNT injections using EMG + US guidance, the incidence of dysphagia fell to 0% [39].

## 13. Practice Guidelines/Recommendations Related to Guidance for BoNT Injections

In 2016, Simpson et al. published a Practice Guideline Update for the American Academy of Neurology [4]. The authors reviewed the literature and made recommendations for the use of BoNTs for blepharospasm, cervical dystonia, adult spasticity and headache. They reported on techniques used to optimize response to BoNT therapy and concluded that “high-volume injections on a BoNT-A and endplate targeting into proximal upper extremity muscles are probably effective strategies for enhancing tone reduction in adult spasticity”. They concluded that comparative studies of three guidance techniques (manual, electrical stimulation, US) did not favor one technique. This is in contrast to the recommendations by other authors based on systematic review of the various guidance techniques [58,59,60,67].

Albanese et al., in 2015, published a consensus statement on the use of BoNTs in the management of cervical dystonia. The authors concluded that while manual guidance alone might be sufficient for some superficial muscles, supplemental guidance with either EMG or US was required for deeper muscles [49,68].

A consensus paper on chemodenervation was published in 2009 by a group of physicians from across Europe [67]. When evaluating guidance and dilution the authors concluded that:

⚬ Muscle end-plate targeting is desirable, but may not be feasible in all muscles. They recommended that injections be placed as near to the motor end-plates as possible, where this information is known, otherwise injection guidance charts should be used.
⚬ In larger muscles where motor point location is ill-defined or diffuse, multiple injections and higher volume injections may be preferable to single site and/or low volume injections.
⚬ In smaller muscles, multiple injection sites and larger volumes may be impractical. When higher doses are needed for small muscles, higher volumes were not required.
⚬ Injection guidance was recommended for deep muscles or where anatomical landmarks were difficult to determine.
⚬ Patient comfort should be considered when choosing an injection volume.

In 2006, Heinen et al. published a European consensus statement on the use of BoNT in children. The authors recommend that “children should receive injections delivered using an accurate localization technique. Classical neurophysiological localization methods (EMG, electrical stimulation) have recently been fine-tuned and amended by sonography which allows precise and painless identification of any target muscle using readily available, non-invasive equipment” [69].

## 14. Summary

Ultrasound guidance is one of several localization techniques available to guide chemodenervation procedures including BoNT injections. US has a number of advantages over other guidance techniques supplemental to manual palpation/surface landmarks which include; direct and continuous visualization of the needle, the target, structures to be avoided and the injectate spread within the target structure. There is Level 1 evidence that instrumented guidance (US, EMG, E-Stim) is superior to injections relying solely on manual guidance. In addition most expert clinicians and professional societies advise against relying solely on anatomic or manual guidance techniques for most BoNT procedures as potentially inaccurate and often less efficacious.

While there is general consensus that ultrasound guidance provides the greatest anatomic accuracy for BoNT injections, additional head-to-head trials are required to resolve whether it is superior to other guidance techniques when used alone. Additional research is also needed to determine when US is best used with an electrophysiological technique.

## Figures and Tables

**Table 1:** Clinical Applications of Botulinum Toxins.

FDA Approved Indications *	Off-Label Uses: Commonly Recommended or Clinically Accepted Applications	Off-Label Uses: Less Commonly Reported or Investigational Applications
Muscle over-activity/imbalance [1] - Blepharospasm ^a,b,c^ - Strabismus ^a^ - Cervical Dystonia ^a,b,c,d^ - Spasticity, upper limb, adults ^a,b,c^ - Spasticity, lower limb, adults ^a,b^ - Spasticity, lower limb, pediatric patients (>2 years of age) ^b^ - Bladder detrusor over-activity in patients with neurologic conditions ^a^ - Overactive bladder ^a^	Muscle over-activity [2,3,6,7,8,9,10,11,12,13] - Focal limb dystonia - Task specific dystonia - Camptocormia - Hemifacial spasm - Spasticity in pediatric patients, upper limb - Bruxism - Palatal tremor - Upper motor neuron syndromes - Schwartz-Jampel syndrome - Parkinson disease related dystonia/rigidity/tremor - Essential tremor - Dystonic tremor - Vocal cord dysfunction - Oromandibular dystonia - Tics - Segmental Myoclonus	Muscle over-activity/imbalance [2,9,10,11,14,15,16,17] - Trigger points - Muscle imbalance associate with obstetrical brachial plexus palsy - Congenital club foot Muscle tightness post hip arthroplasty - Facial paralysis/imbalance - Restless leg syndrome - Myokymia - Myoclonus - Synkinesis - Bell’s Palsy
Neurosecretory dysfunction [1] - Primary axillary hyperhidrosis ^a^	Neurosecretory dysfunction [5,9,16,17] - Sialorrhea - Hyperhidrosis, palmar/plantar - Frey syndrome (gustatory sweating)	Neurosecretory dysfunction [9,10,11] - Prostatic hypertrophy - Hyperlacrimation
Pain conditions [1,9] - Chronic migraine ^a^ - Pain associated with cervical dystonia ^a,b,c^	Pain conditions [6,7,10,13,18] - Plantar fasciitis - Lateral epicondylitis/epicondylosis - Postoperative pain/spasms in cerebral palsy - Occipital neuralgia - Piriformis syndrome - Postoperative pain associated with total knee arthroplasty - Pain associated with upper motor neuron syndrome	Pain conditions [9,10,11,13,17,18,19] - First bite syndrome - Trigger points - Myofascial pain - Fibromyalgia - Lateral epicondylitis or epicondylosis - Joint pain associated with osteoarthritis - Chronic daily headache - Tension type headache - Cervicogenic headache - Patellofemoral pain syndromes - Temporo-mandibular joint pain Post herpetic neuralgia - Trigeminal neuralgia - Neuropathic pain - Chronic pelvic pain - Alodynia associated with diabetic neuropathy - Dyspareunia - Post-surgical pain syndromes
Urologic/Gynecologic [1] - Bladder detrusor over-activity in patients with neurologic conditions ^a^ - Overactive bladder ^a^	Urologic/Gynecologic [9,10,11] - Sphincter dyssenergy - Bladder outlet obstruction - Bladder spasms/pain - Urge incontinence	Urologic/Gynecologic [10,11] - Prostatic hypertrophy - Chronic pelvic pain - Dyspareunia - Pelvic floor pain - Vaginismus - Hot flashes
Gastrointestinal	Gastrointestinal [9,10,11] - Achalasia - Rectal fissure	Gastrointestinal [9,10,17] - Morbid obesity - Intractable constipation - Neurogenic dysphagia - Diabetic gastroparesis
Ophthalmologic uses [1,12] ⚬ Strabismus ^a^ ⚬ Blepharospasm ^a,b,c^	Off label Ophthalmologic applications [2,9,10,11,17] - Apraxia of eyelid opening - Ptosis - Acquired disruption of motor fusion (intractable diplopia) - Benign eyelid fasciculation - Nystagmus - Tear film conditions - Oscillopsia - Corneal astigmatism	
Dermatologic	Off label dermatologic [11,19,20,21] - Seborrhea - Keloids/hypertrophic scars - Linear bullous dermatitis - Hidradinitis supporativa - Alopecia - Psoriasis - Facial flushing - Oily skin	
Aesthetic/Cosmetic - Moderate to severe frown/glabellar lines ^a,b,c^ - Moderate to severe lateral canthal lines ^a^ - Moderate to severe forehead lines ^a^	Off label Aesthetic/Cosmetic Applications [11,19,20,21] - Platysmal bands - Depressed brow - Hypertrophic orbicularis oculi muscle (small palpebral aperture) - Rhytides from upper nasalis muscle contraction (bunny lines) - Nostril flare - Drooping nasal tip - Nasolabial folds (selected patients) - Vertical perioral rhytides - Mouth frown - Gummy smile - Melomental folds (marionette lines) - Mental crease (horizontal crease on the chin) - Peau d’orange chin - Masseteric hypertrophy (square jaw) - Horizontal neck lines	
Other uses	- Raynauds [6] - Major Depression [11]	

* Federal Drug Administration (FDA) Approved indications in November 2017. ^a^ OnabotulinumtoxinA. ^b^ AbobotulinumtoxinA. ^c^ IncobotulinumtoxinA. ^d^ RimabotulinumtoxinB.

**Table 2:** Guidance methods for chemodenervation procedures.

Guidance Method	Useful to:	Required Equipment	Required Training	Recognized or Possible Limitations:
Anatomic Guidance [22,24,25]	- Identify muscle location/site for needle insertion based on; landmarks, palpation, passive or active Range of Motion (ROM)	Anatomic atlases or reference guides Injection supplies including hypodermic needles of various sizes	Gross anatomy lab training and education in medical school and or refresher courses during or after post-graduate training	- Cannot distinguish anatomic variations or rearrangements - Contractures, deformity - Cannot assess muscle depth or atrophy - Difficulty positioning patients as described in reference guides
Electromyography (EMG) [22,24,25]	- Provides auditory and or vision feedback of muscle activity - May help to Identify muscles contributing to a posture or position and level of activity of possible target muscle - May be more useful in patients with focal dystonia than those with spasticity or dystonia associated with UMNS - EMG can assist with motor point localization	EMG amplifier or electrodiagnostic instrument Injection supplies including insulated needle electrode for injection	Electrodiagnostic training during residency and or BoNT injection training courses	- Equipment availability/cost - Only useful for muscle targets - Difficult to position patients as described - Difficult to determine depth and location of muscle. - Co-contraction/mass synergy limit muscle isolation - Teflon coated needles are expensive and more painful to insert - May increase procedure time for clinicians inexperienced with this technique
Electrical Stimulation (E-Stim) [22,24,25]	- Provides visible feedback of muscle contraction in target muscle - May be more useful than EMG in patients with Upper Motor Neuron Syndromes - E-Stim can assist with motor point localization	Portable electrical stimulator or electrodiagnostic instrument Injection supplies including insulated needle electrode for injection	Electrodiagnostic training during residency and or BoNT injection training courses	- Equipment availability/cost - Only useful for muscle targets - Painful to perform, requires sedation in most children. - Difficult to position patients as described - Difficult to determine depth and location of muscle - EMG assists with motor point localization. - Teflon coated needles are expensive and more painful to insert - May increase procedure time for clinicians inexperienced with this technique
Ultrasound [22,24,25]	- Precise localization of muscle position, depth, size - Continuous visualization of needle, target and structures to be avoided - Visualization of injectate within target muscle/structure - Useful for non-muscle targets - May provide information related to muscle activity and hypertrophy or atrophy - Accessibility/availability - Ease of scheduling	Ultrasound machine Transducers of various frequency Injection supplies including hypodermic needles of various sizes and insulated needle electrode for injection for US + EMG or US + E-Stim combined guidance	Ultrasound training during post-graduate training and or for practicing clinicians including ultrasound: Physics/instrumentation Properties of relevant tissues Pattern recognition Hands on practice for target identification Procedural guidance skills practice	- Equipment availability/cost - Requires trained MD/sonographer - Time/training commitment - May increase procedure time for clinicians inexperienced with this technique
Other imaging techniques [24,25]: - Fluoroscopy - CT - MRI	- Useful to identify some structures but not muscle targets - Useful to identify many muscles/targets - Useful to identify many muscles/targets	Fluoroscopy, MRI or CT equipment, radiology personnel including technicians and radiologist	Referral to interventional radiologist for the BoNT procedure	- CT/Fluoroscopy: exposure to ionizing radiation - Fluoroscopy: Does not visualized muscles/targets or activity of muscles - Cost - Limited access - Time consuming - Scheduling difficulties
Combinations of Techniques [24,25] - EMG + US - EMG + E-Stim	- EMG + US is useful to precisely locate target while providing information about muscle activity. EMG also assists with motor point localization. - E-Stim combined with US is useful to precisely locate target, place needle in target (muscles) or adjacent to target (nerves) for motor point localization or nerve blocks	See above	Combinations of the above training	- Combined EMG-US is only useful for muscle targets - Combined EMG-US is only useful for muscle and or nerve targets

**Table 3:** Ultrasound Transducer Frequency, Depth of Penetration and Applications.

Frequency in MHz	Penetration Depth	Clinical Application
3–1	12–22 cm	OB/GYN
5–3	12–15 cm	Abdomen and or deep muscles (piriformis, iliopsoas) or obese patients [52,53]
7.5–5	8–12 cm	Moderately deep muscles (thigh, hip), larger muscular patients [52,53]
12–5	3.5–12 cm	General imaging including; neck, limb, trunk muscles BoNT Injections
		Smaller children: Most muscles including hip region
		Small adults: Superficial and moderately deep muscles
		Large or obese patients: Superficial limb, neck, trunk muscles [52,53]
17–5	2–10 cm	Superficial muscles of hand, neck, limbs [5,52]

## Supplementary Materials

The following are available online at www.mdpi.com/2072-6651/10/1/18/s1, Video S1: Long-axis B-mode US Cine-loop Digit Isolation Injection US and EMG, Video S2: US and ESTIM Guided Interscalene Nerve Block.
